# Shorter epinephrine dosing intervals and one-year survival after in-hospital pediatric cardiopulmonary resuscitation^☆^

**DOI:** 10.1016/j.jped.2026.101520

**Published:** 2026-03-05

**Authors:** Bruno Marcelo Herculano Moura, Edison Ferreira de Paiva, Ivan Peres Costa, Thomaz Bittencourt Couto, Cláudio Schvartsman, Tania Miyuki Shimoda Sakano, Amelia Gorete Reis

**Affiliations:** a Instituto da Criança e do Adolescente do Hospital das Clínicas da Faculdade de Medicina da Universidade de São Paulo, São Paulo, SP, Brazil; b Universidade Evangélica de Goiás, UniEvangélica, Anápolis, GO, Brazil; c Einstein Hospital Israelita, São Paulo, SP, Brazil

**Keywords:** Epinephrine, Cardiopulmonary resuscitation, Child, In-hospital cardiac arrest, Pediatric

## Abstract

**Objective:**

To evaluate the association between the interval doses of epinephrine and one-year survival and one-year neurological prognosis after pediatric in-hospital cardiac arrest (IHCA).

**Methods:**

This observational retrospective cohort study included pediatric patients (0–18 years) who experienced IHCA and received at least two doses of epinephrine from January 2015 to December 2022. Data were collected following the Utstein style. The mean interval between epinephrine doses was categorized as 〈 3, 3–5, or > 5 min. Primary outcome was one-year survival; secondary outcomes were survival to hospital discharge and one-year neurological prognosis, assessed by the Pediatric Cerebral Performance Category.

**Results:**

194 patients were eligible. In the univariate adjusted analysis, patients who received epinephrine at intervals shorter than 3 min had a 2.3-fold increased chance of one-year survival (OR 2.3; 95 % CI 1.0–5.5; *p* = 0.042), although this association was not sustained in the multivariable regression. Longer intervals between epinephrine doses (OR: 0.71; 95 % CI 0.52–0.71; *p* = 0.03), continuous vasoactive drugs infusion prearrest (OR 0.1; 95 % CI 0.1–0.4; *p* < 0.001), longer resuscitation duration (OR 0.8; 95 % CI 0.7–0.9; *p* = 0.028), epinephrine doses (OR 0.68; 95 % CI 0.56–0.81; *p* < 0.001) were associated with reduced one-year survival. Neurological deterioration was observed in 6 (16.6 %) of the 36 patients after one year (*p* = 0.001). No association was found between epinephrine dosing intervals (OR 0.72; 95 % CI 0.16–3.14; *p* = 0.65) and neurological outcomes.

**Conclusion:**

Epinephrine dosing interval was not independently associated with one-year survival after adjusted analyses. These findings underscore the complexity of pediatric resuscitation and support further multicenter prospective studies.

## Introduction

Cardiac arrest (CA) is a significant cause of morbidity and mortality in the pediatric population worldwide [1]. Increasing survival rates of in-hospital pediatric CA patients have been reported in high-income countries [2,3], and this trend has also been demonstrated in a limited number of studies in middle-income countries [4,5,6]. The population experiencing in-hospital cardiac arrest (IHCA) consists mainly of patients with severe comorbidities, representing 71 % to 90.9 % of cases [4,7,8]. Although the prognosis of in-hospital pediatric cardiopulmonary resuscitation (CPR) can be influenced by many factors [6], the role of epinephrine remains highly debated. Epinephrine administration during CPR increases coronary and cerebral perfusion [9], but it also stimulates cardiac adrenergic receptors, potentially leading to harmful effects on the heart during the ischemia and reperfusion phases [10]. The interval of epinephrine administration may play a crucial role in balancing its desirable and adverse effects, thus impacting outcomes. The International Liaison Committee on Resuscitation (ILCOR) 2023[11] recommends administering epinephrine at 3- to 5-minute intervals; however, recent literature[12,13] challenges this recommendation, suggesting that intervals shorter than 3 min are associated with increased chances of return of spontaneous circulation (ROSC) and survival to hospital discharge.

There remains an inconsistency in the literature regarding the epinephrine dose interval and CPR prognosis. Hoyme et al. (2017)[1] in a multicenter retrospective cohort study demonstrated that, in adjusted analyses, administering epinephrine at intervals longer than 5 min was significantly associated with improved hospital survival. In contrast, Kienzle et al. (2021)[12] in a single-center retrospective cohort study reported that epinephrine doses at intervals <2 min were associated with increased chances of survival and better neurological outcomes. A recent multicenter cohort study by Kienzle et al. (2024)[13] concluded that shorter dose intervals (<3 min) led to increased ROSC but were not associated with favorable neurological outcomes. Studies in adults have also shown inconsistent results regarding the epinephrine dose interval[14,15] and CPR prognosis [15].

Most pediatric CPR studies have been conducted in high income countries [1,12,13], with few scientific studies in middle[4, 6]income nations. Studies from middle-income countries are needed to better understand the epinephrine dosing during CPR, as there are gaps in the scientific evidence supporting current guidelines. Another important aspect is the lack of pediatric studies evaluating the role of epinephrine in long-term survival and neurological outcomes.

The hypothesis for this study was that shorter epinephrine dose intervals than currently recommended are associated with better pediatric CPR outcomes. Therefore, the objective of this study was to evaluate the associations of the interval between epinephrine doses administered during in-hospital pediatric CPR and one-year survival and one-year neurological prognosis.

## Material and methods

### Study design and study location

This was a retrospective observational cohort study of pediatric patients who underwent CPR from January 2015 to December 2022 and received at least two doses of epinephrine. Data were obtained from the local institutional registry, which follows Utstein style [16]. The project was approved by the hospital's ethics committee. Due to the inability to obtain informed consent at the time of cardiac arrest, presumed consent was applied, consistent with the approach used in prior resuscitation studies. The study was approved with a waiver of informed consent by the local Ethics and Research Committee, under protocol number: 29,404,620.0.0000.0068, on March 5th, 2020, and was conducted in a public university hospital with tertiary and quaternary healthcare, which is located in a large urban center and specializes in treating pediatric patients with complex chronic diseases. Extracorporeal cardiopulmonary resuscitation (ECPR) was not used in any of the included cardiac arrest events.

### Study participants

Patients aged 0 to 18 years who experienced IHCA, regardless of duration, and who received at least two doses of epinephrine were included. Patients with out-of-hospital cardiac arrest (OHCA), CA due to trauma, or those who received boluses of other vasoactive drugs (e.g. vasopressin) instead of epinephrine during CPR were excluded.

### Primary and secondary outcomes

The variables of interest are presented in Table 1. The interval between epinephrine doses was the exposure and the independent variable of interest. The interval between doses was defined as the time between the first dose of epinephrine and the outcome of resuscitation (ROSC or death), divided by the total number of epinephrine doses administered after the first dose, and was categorized as < 3 min, 3–5 min, and > 5 min. In the event of doubts, inconsistencies, or unavailable data, medical records were analyzed, and interviews were conducted with healthcare professionals involved in patient care or with family members.

**Table 1:** Patient characteristics.

Variables	Epinephrine average dosing period				p-value^2^
	Overall, *n* = 194^1^	< 3 min, *n* = 87^1^	3–5 min, *n* = 90^1^	> 5 min, *n* = 17^1^	
Time of day of arrest					0.121
Day	95 (48.97 %)	48 (55.17 %)	37 (41.11 %)	10 (58.82 %)	
Night	99 (51.03 %)	39 (44.83 %)	53 (58.89 %)	7 (41.18 %)	
Time of week of arrest					0.009
Weekend	54 (27.84 %)	15 (17.24 %)	32 (35.56 %)	7 (41.18 %)	
Weekday	140 (72.16 %)	72 (82.76 %)	58 (64.44 %)	10 (58.82 %)	
Sex					0.595
Female	98 (50.52 %)	41 (47.13 %)	49 (54.44 %)	8 (47.06 %)	
Male	96 (49.48 %)	46 (52.87 %)	41 (45.56 %)	9 (52.94 %)	
Age group					0.828
Neonate, < 1 mo	51 (26.29 %)	23 (26.44 %)	23 (25.56 %)	5 (29.41 %)	
Infant, 1–12 mo	69 (35.57 %)	32 (36.78 %)	30 (33.33 %)	7 (41.18 %)	
Child, 1–12 yo	58 (29.90 %)	27 (31.03 %)	28 (31.11 %)	3 (17.65 %)	
Adolescent, > 12 yo	16 (8.25 %)	5 (5.75 %)	9 (10.00 %)	2 (11.76 %)	
Preexisting conditions	186 (95.88 %)	83 (95.40 %)	88 (97.78 %)	15 (88.24 %)	0.154
Genetic	94 (48.45 %)	44 (50.57 %)	45 (50.00 %)	5 (29.41 %)	0.258
Hepatic	45 (23.20 %)	23 (26.44 %)	18 (20.00 %)	4 (23.53 %)	0.624
Neurologic	31 (15.98 %)	10 (11.49 %)	18 (20.00 %)	3 (17.65 %)	0.282
Other diseases	81 (41.75 %)	36 (41.38 %)	39 (43.33 %)	6 (35.29 %)	0.823
Location of arrest					0.001
PICU	160 (82.47 %)	72 (82.76 %)	74 (82.22 %)	14 (82.35 %)	
OR	10 (5.15 %)	10 (11.49 %)	0 (0.00 %)	0 (0.00 %)	
Pedriatric Floor with telemetry	5 (2.58 %)	0 (0.00 %)	4 (4.44 %)	1 (5.88 %)	
Emergency department	19 (9.79 %)	5 (5.75 %)	12 (13.33 %)	2 (11.76 %)	
Arrest witnessed	191 (98.45 %)	86 (98.85 %)	88 (97.78 %)	17 (100.00 %)	>0.999
Previous insertion of airway	141 (72.68 %)	74 (85.06 %)	54 (60.00 %)	13 (76.47 %)	<0.001
Insertion of airway during CPR	40 (20.62 %)	10 (11.49 %)	25 (27.78 %)	5 (29.41 %)	0.012
Previous cardiac monitoring	151 (77.84 %)	75 (86.21 %)	67 (74.44 %)	9 (52.94 %)	0.006
Prearrest vasoactive infusion	104 (53.61 %)	51 (58.62 %)	45 (50.00 %)	8 (47.06 %)	0.44
Calcium during CPR	80 (41.24 %)	27 (31.03 %)	46 (51.11 %)	7 (41.18 %)	0.025
Bicarbonate during CPR	125 (64.43 %)	52 (59.77 %)	66 (73.33 %)	7 (41.18 %)	0.019
Immediate cause					
Respiratory decompensation	83 (42.78 %)	36 (41.38 %)	40 (44.44 %)	7 (41.18 %)	0.91
Metabolic decompensation	22 (11.34 %)	9 (10.34 %)	12 (13.33 %)	1 (5.88 %)	0.743
Shock	83 (42.78 %)	39 (44.83 %)	37 (41.11 %)	7 (41.18 %)	0.874
Initial rhythm					
Pulseless electrical activity	51 (26.29 %)	20 (22.99 %)	27 (30.00 %)	4 (23.53 %)	0.556
Asystole	41 (21.13 %)	16 (18.39 %)	21 (23.33 %)	4 (23.53 %)	0.701
Bradycardia*	91 (46.91 %)	46 (52.87 %)	37 (41.11 %)	8 (47.06 %)	0.293
VF/VT	7 (3.61 %)	3 (3.45 %)	3 (3.33 %)	1 (5.88 %)	0.711
Missing	4	2	2	0	
Duration of CPR, min					<0.001
Mean (SD)	18.29 (15.24)	9.67 (9.26)	24.01 (14.21)	32.12 (20.68)	
Median (IQR)	14.50 (7.00, 25.00)	6.00 (5.00, 11.00)	20.00 (14.00, 30.00)	26.00 (20.00, 35.00)	
Range	0.00, 95.00	0.00, 60.00	7.00, 77.00	11.00, 95.00	
Time to 1st epinephrine dose, min				<0.001	
Mean (SD)	1.71 (2.27)	0.72 (1.13)	2.11 (2.15)	4.59 (3.78)	
Median (IQR)	1.00 (0.00, 3.00)	0.00 (0.00, 1.00)	2.00 (0.00, 4.00)	5.00 (3.00, 6.00)	
Range	0.00, 13.00	0.00, 5.00	0.00, 12.00	0.00, 13.00	
Epinephrine mean dosing period, min				<0.001	
Mean (SD)	3.23 (1.49)	1.99 (0.73)	3.87 (0.60)	6.20 (1.36)	
Median (IQR)	3.17 (2.00, 4.00)	2.00 (1.50, 2.55)	3.75 (3.35, 4.30)	5.50 (5.20, 7.00)	
Range	0.00, 9.00	0.00, 3.00	3.00, 5.00	5.00, 9.00	
Total epinephrine doses				<0.001	
Mean (SD)	4.01 (2.21)	3.21 (1.94)	4.73 (2.23)	4.29 (2.05)	
Median (IQR)	3.00 (2.00, 5.00)	2.00 (2.00, 4.00)	4.00 (3.00, 6.00)	4.00 (3.00, 5.00)	
Range	2.00, 10.00	2.00, 10.00	2.00, 10.00	2.00, 10.00	
ROSC	109 (56.19 %)	58 (66.67 %)	43 (47.78 %)	8 (47.06 %)	0.03
Survival to discharge	35 (18.04 %)	21 (24.14 %)	12 (13.33 %)	2 (11.76 %)	0.156
30 days	42 (21.65 %)	25 (28.74 %)	15 (16.67 %)	2 (11.76 %)	0.099
180 days	33 (17.01 %)	21 (24.14 %)	11 (12.22 %)	1 (5.88 %)	0.054
1 year	31 (16.06 %)	20 (22.99 %)	10 (11.24 %)	1 (5.88 %)	0.062
Baseline PCPC					0.324
1	22 (61.11 %)	14 (66.67 %)	7 (50.00 %)	1 (100.00 %)	
2	9 (25.00 %)	3 (14.29 %)	6 (42.86 %)	0 (0.00 %)	
3	5 (13.89 %)	4 (19.05 %)	1 (7.14 %)	0 (0.00 %)	
4	0 (0.00 %)	0 (0.00 %)	0 (0.00 %)	0 (0.00 %)	
One-year survival PCPC					0.827
1	15 (41.67 %)	8 (38.10 %)	6 (42.86 %)	1 (100.00 %)	
2	10 (27.78 %)	7 (33.33 %)	3 (21.43 %)	0 (0.00 %)	
3	10 (27.78 %)	6 (28.57 %)	4 (28.57 %)	0 (0.00 %)	
4	1 (2.78 %)	0 (0.00 %)	1 (7.14 %)	0 (0.00 %)	
Categorization of Baseline PCPC			0.679		
1 + 2	31 (86.11 %)	17 (80.95 %)	13 (92.86 %)	1 (100.00 %)	
3 + 4	5 (13.89 %)	4 (19.05 %)	1 (7.14 %)	0 (0.00 %)	
Categorization of One-year survival PCPC			0.806		
1 + 2	25 (69.44 %)	15 (71.43 %)	9 (64.29 %)	1 (100.00 %)	
3 + 4	11 (30.56 %)	6 (28.57 %)	5 (35.71 %)	0 (0.00 %)	

1 n ( %).

2 Pearson’s Chi-squared test; Fisher’s exact test; Kruskal-Wallis rank sum test; mo, month; yo, years old; PICU, pediatric intensive care unit; OR, operating room; CPR, Cardiopulmonary resuscitation; VF, ventricular fibrilatrion; VT, ventricular tachycardia; SD, Standard deviation; IQR, interquartile range; min, minutes; ROSC, return of spontaneous circulation; PCPC, Pediatric Cerebral Performance Category. (Baseline event characteristics by exposure to CPR and one-year survival). Data are shown as n ( %), mean (SD) or median (interquartile range). *Bradycardia with poor perfusion.

The primary outcome was one-year survival. Secondary outcomes were survival to hospital discharge and neurological status at one-year. Neurological status was assessed by the Pediatric Cerebral Performance Scale (PCPC) [17], following the P-COSCA recommendation [18], which defines favorable outcomes as a PCPC score of 1 or 2 and unfavorable outcomes (neurological disability, persistent vegetative state) as a PCPC score of 3, 4, or 5.

### Statistical analysis

For qualitative variables, absolute frequencies (n) and relative frequencies ( %) were reported, whereas measures of central tendency and dispersion, including the mean, median, interquartile range, range, and standard deviation, were provided for quantitative variables. Associations between demographic and clinical variables (categorical variables) and outcomes (survival to discharge and one-year survival) were evaluated using Fisher’s exact test or the chi-square test, as appropriate. Comparisons of continuous variables between outcome groups were performed using Student’s *t*-test or the nonparametric Mann‒Whitney U test, depending on data distribution, which was assessed with the Shapiro‒Wilk test. McNemar’s test was applied to evaluate changes in the PCPC scale score pre-arrest and after one-year. To assess the effect of the average interval between doses on outcomes (hospital discharge and one-year survival), an univariable logistic regression model was fitted with results reported as odds ratios (ORs) and 95 % confidence intervals (CIs). Unadjusted ORs (uOR) were also estimated for demographic and clinical factors. Multivariable logistic regression models were fitted using the categorized dose interval (< 3 min, 3–5 min, > 5 min) and known risk factors and confounders, including total epinephrine doses, prearrest continuous vasoactive drug infusion, and CPR duration (in minutes). Data analyses were conducted using the R package version 4.4.1 with two-sided tests at a significant level of 0.05.

## Results

**Fig. 1:**
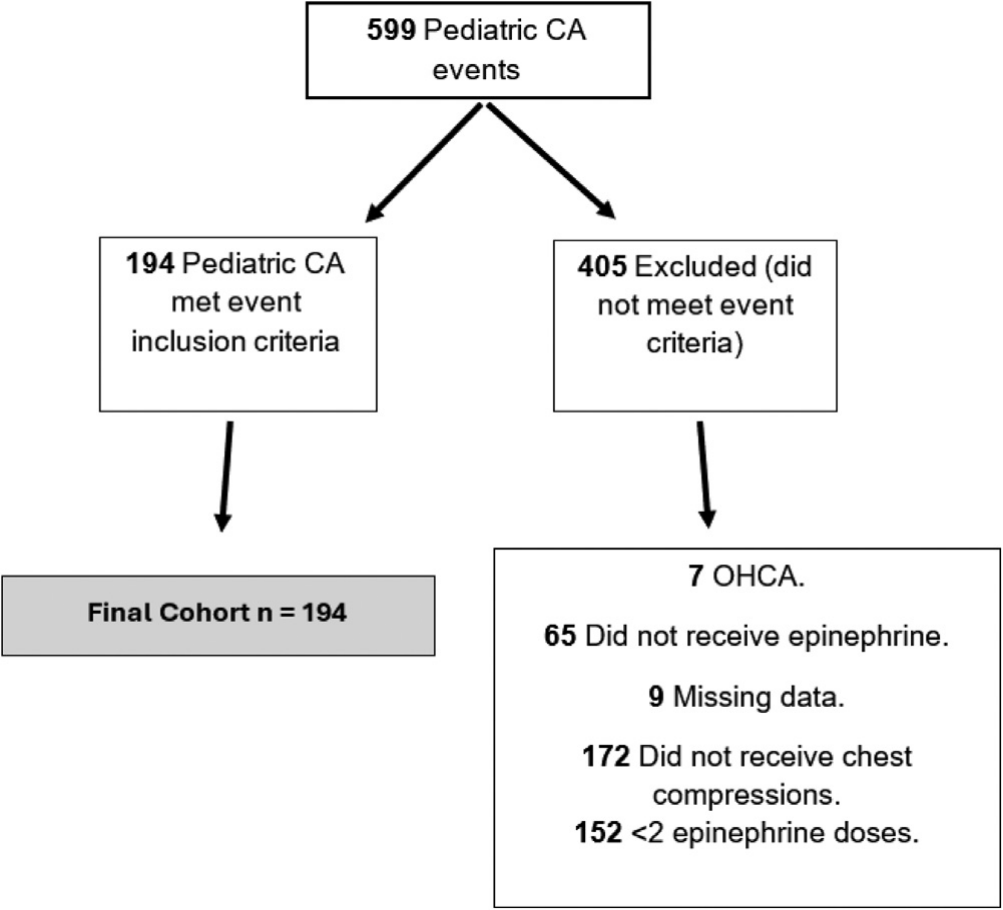
Patient flowchart for study. CA, cardiac arrest; OHCA, out of hospital cardiac arrest.

A total of 599 patients were included in the CPR registry, and after applying the exclusion criteria, a final sample of 194 eligible patients was obtained (Figure 1). The descriptive events analysis is presented in Table 1. CPR events occurred almost equally by sex (female 50.52 %) and the predominant age group was 1–12 months (35.57 %). Most patients had a preexisting condition (95.88 %). The most observed initial rhythm was bradycardia (47.89 %) and more than half of the population was receiving continuous vasoactive drug infusion prearrest (53.6 %). The mean interval between epinephrine doses administered during CPR was 3.23 min, 46.39 % of patients received epinephrine at an interval of 3–5 min. The mean epinephrine doses were 2.69, the mean CPR duration was 18.29 min. In terms of outcomes, ROSC occurred in 56.19 % of patients, 18.04 % were discharged, and 16.06 % were alive at one-year.

### One-Year survival status

In the univariable analysis, a statistically significant association was observed between longer mean epinephrine intervals and one-year survival (OR: 0.71; 95 % CI 0.52–0.95; *p* = 0.02), as demonstrated in Table 2. Specifically, patients who received epinephrine at intervals of <3 min had a 2.3-fold higher chance of one-year survival compared to those with intervals of 3–5 min (OR: 2.36; 95 % CI 1.05–5.58; *p* = 0.042). Furthermore, a higher number of epinephrine doses OR: 0.67; 95 % CI 0.49–0.86; *p* = 0.006) as well as the administration of continuous vasoactive drugs prior CA and longer CPR duration were inversely associated with one-year survival (OR: 0.23; 95 % CI 0.09–0.54; *p* = 0.001; OR: 0.90; 95 % CI 0.85–0.95; *p* < 0.001, respectively). In the multivariable analysis, prearrest continuous vasoactive infusion (OR = 0.18; 95 % CI, 0.06–0.43; *p* < 0.001) and longer CPR duration (OR = 0.87; 95 % CI, 0.77–0.97; *p* = 0.02) were significantly associated with reduced one-year survival. In contrast, total epinephrine doses, the epinephrine mean dosing interval, and epinephrine dosing intervals (< 3 min, 3–5 min, > 5 min) were not significantly associated with one-year survival.

**Table 2:** One-year survival univariable and multivariable analysis.

	One-year survival				Univariable model				Multivariable model		
	Overall, *n* = 193^1^	No, *n* = 162^1^	Yes, *n* = 31^1^	p-value^2^	n	OR^3^	95 % CI^3^	p-value	OR^3^	95 % CI^3^	p-value
Total epinephrine doses				<0.001	193	0.673	0.490 - 0.867	0.006	1.076	0.672 - 1.677	0.752
Mean (SD)	4.02 (2.21)	4.22 (2.23)	2.97 (1.76)								
Median (IQR)	3.00 (2.00 −5.00)	3.50 (2.25 - 5.00)	2.00 (2.00 - 3.00)								
Range	2.00 - 10.00	2.00 - 10.00	2.00 - 10.00								
Previous vasoactive infusion				<0.001	193						
No	89 (46.11 %)	66 (40.74 %)	23 (74.19 %)			Ref			Ref		
Yes	104 (53.89 %)	96 (59.26 %)	8 (25.81 %)			0.239	0.095 - 0.547	0.001	0181	0.068 - 0.436	<0.001
Duration of CPR, min				<0.001	193	0.907	0.853 - 0.953	<0.001	0879	0.776 - 0.975	0.028
Mean (SD)	18.32 (15.28)	20.02 (15.78)	9.42 (7.73)								
Median (IQR)	15.00 (7.00 - 25.00)	16.00 (9.00 - 27.00)	7.00 (4.50 - 10.50)								
Range	0.00 - 95.00	0.00 - 95.00	2.00 - 35.00								
Epinephrine mean dosing interval, min				0.013	193	0.714	0.521 - 0.952	0.028			
Mean (SD)	3.22 (1.49)	3.32 (1.43)	2.68 (1.67)								
Median (IQR)	3.14 (2.00, 4.00)	3.31 (2.50, 4.11)	2.25 (1.50 - 3.58)								
Range	0.00 - 9.00	0.50 - 9.00	0.00 - 8.00								
Categorization of epinephrine interval dosing				0.062	193						
3–5min	89 (46.11 %)	79 (48.77 %)	10 (32.26 %)			Ref			Ref		
< 3min	87 (45.08 %)	67 (41.36 %)	20 (64.52 %)			2.358	1.053 - 5.581	0.042	0.726	0.219 - 2.406	0.598
> 5min	17 (8.81 %)	16 (9.88 %)	1 (3.23 %)			0.494	0.026 - 2.861	0.515	0.833	0.039 - 6.489	0.878

1 n, population number ( %).

2 Pearson’s Chi-squared test; Fisher’s exact test; Wilcoxon rank sum test.

3 OR, Odds Ratio; CI, confidence interval; min, minutes; OR, odds ratio; CPR, cardiopulmonar resuscitation; SD, Standart deviation; IQR, interquartile range.

### Survival to discharge

There were no statistically significant associations between epinephrine dose intervals (reference 3–5 min) and survival to hospital discharge in either the univariate or multivariate analysis (uOR: 0.56; 95 % CI 0.17–1.80; *p* = 0.33 and OR: 1.58; 95 % CI 0.19–9.42; *p* = 0.62, respectively). In the univariable analysis, patients who received a higher number of epinephrine doses had a lower likelihood of survival to hospital discharge (OR: 0.63; 95 % CI 0.46–0.82; *p* = 0.002). Another important finding was that the mean dose interval was not associated with survival to discharge (OR: 0.82; 95 % CI 0.62–1.06; *p* = 0.148) according to the univariable analysis. In the multivariable analysis, longer CPR duration was also statistically significantly associated with a reduced likelihood of survival to discharge (OR: 0.87; 95 % CI 0.77–0.96; *p* = 0.016), as well as continuous vasoactive drugs infusion prior to CPR (OR: 0.17; 95 % CI 0.06–0.40; *p* < 0.001) as demonstrated in Table 3.

**Table 3:** Survival to discharge univariable and multivariable analysis.

	Survival to discharge				Univariable model				Multivariable model		
	Overall, *n* = 194^1^	No, *n* = 159^1^	Yes, *n* = 35^1^	p-value^2^	n	OR^3^	95 % CI^3^	p-value	OR^3^	95 % CI^3^	p-value
Total epinephrine doses				<0.001	194	0.634	0.460 - 0.820	0.002	1.014	0.639 - 1.560	0.95
Mean (SD)	4.01 (2.21)	4.26 (2.24)	2.89 (1.68)								
Median (IQR)	3.00 (2.00 - 5.00)	4.00 (3.00 - 5.00)	2.00 (2.00 - 3.00)								
Range	2.00 - 10.00	2.00 - 10.00	2.00 - 10.00								
Previous vasoactive infusion				<0.001	194						
No	90 (46.39 %)	64 (40.25 %)	26 (74.29 %)			Ref			Ref		
Yes	104 (53.61 %)	95 (59.75 %)	9 (25.71 %)			0.233	0.098 - 0.513	0.001	0.176	0.069 - 0.409	<0.001
Duration of CPR, min				<0.001	194	0.908	0.858 - 0.951	<0.001	0.872	0.774 - 0.965	0.016
Mean (SD)	18.29 (15.24)	20.20 (15.86)	9.60 (7.48)								
Median (IQR)	14.50 (7.00 - 25.00)	16.00 (9.00 - 27.50)	7.00 (4.50 - 11.50)								
Range	0.00 - 95.00	0.00 - 95.00	2.00 - 35.00								
Epinephrine mean dosing interval, min				0.038	194	0.82	0.620 - 1.062	0.148			
Mean (SD)	3.23 (1.49)	3.30 (1.36)	2.90 (1.96)								
Median (IQR)	3.17 (2.00 - 4.00)	3.29 (2.50 - 4.07)	2.50 (1.50 - 3.71)								
Range	0.00 - 9.00	0.50 - 8.50	0.00 - 9.00								
Categorization of epinephrine dosing interval				0.156	194						
3–5min	90 (46.39 %)	78 (49.06 %)	12 (34.29 %)			Ref			Ref		
< 3min	87 (44.85 %)	66 (41.51 %)	21 (60.00 %)			2.068	0.960 - 4.634	0.068	0.568	0.176 - 1.808	0.339
> 5min	17 (8.76 %)	15 (9.43 %)	2 (5.71 %)			0.867	0.127 - 3.622	0.86	1.588	0.190 - 9.424	0.629

1 n, population number ( %).

2 Pearson’s Chi-squared test; Fisher’s exact test; Wilcoxon rank sum test.

3 OR, Odds Ratio; CI, confidence interval; min, minutes; OR, odds ratio; CPR, cardiopulmonar resuscitation; SD, Standart deviation; IQR, interquartile range.

### One-year survival neurologic status outcome

Among the 36 patients who were alive after one-year CPR, the PCPC score of 6 patients changed from favorable (PCPC score of 1 or 2) to unfavorable (PCPC score of 3 or 4), indicating that only 16.6 % of survivors experienced worsened neurological status after one-year (*p* = 0.01). No association was found between the epinephrine doses and neurological prognosis at one year (Table 4), but an association was found between fewer epinephrine doses and favorable neurological outcomes at one year (OR: 2.79; 95 % CI 1.26–8.70; *p* = 0.02).

**Table 4:** One-year survival neurological status outcome, univariable and multivariable analysis.

	One-year survival PCPC				Univariable model				Multivariable model			
	Overall, *n* = 36^1^	1 + 2, *n* = 25^1^	3 + 4, *n* = 11^1^	p-value^2^	n	OR^3^	95 % CI^3^	p-value	n	OR^3^	95 % CI^3^	p-value
Previous vasoactive infusion				0.4	36							
No	29 (80.56 %)	19 (76.00 %)	10 (90.91 %)			—	—		Ref	—	—	
Yes	7 (19.44 %)	6 (24.00 %)	1 (9.09 %)			0.317	0.016 - 2.227	0.317	0.045	0,05	0.000, 0.914	0.125
Total epinephrine doses				0.487	36	1.445	0.966 - 2.440	0.102	2.907	2.793	1.260, 8.702	0.029
Mean (SD)	3.00 (1.79)	2.64 (0.95)	3.82 (2.82)									
Median (IQR)	2.00 (2.00 - 3.00)	2.00 (2.00 - 3.00)	2.00 (2.00 - 5.00)									
Range	2.00 - 10.00	2.00 - 5.00	2.00 - 10.00									
Duration of CPR, min				0.797	36	1.022	0.928 - 1.121	0.632	0.829	0.836	0.619, 1.044	0.158
Mean (SD)	10.08 (7.68)	9.68 (6.71)	11.00 (9.85)									
Median (IQR)	7.50 (5.00 - 12.00)	7.00 (5.00 - 12.00)	8.00 (5.00 - 10.50)									
Range	2.00 - 35.00	2.00 - 25.00	3.00 - 35.00									
Time to first epinephrine dose				0.053	36	0.542	0.204 - 0.964	0.105				
Mean (SD)	1.28 (2.12)	1.68 (2.38)	0.36 (0.92)									
Median (IQR)	0.00 (0.00 - 2.00)	1.00 (0.00 - 3.00)	0.00 (0.00 - 0.00)									
Range	0.00 - 10.00	0.00 - 10.00	0.00 - 3.00									
Epinephrine mean dosing interval, min				0.705	36	0.874	0.513 - 1.381	0.582	1.051			
Mean (SD)	2.82 (1.58)	2.91 (1.68)	2.60 (1.40)									
Median (IQR)	2.50 (1.50 - 3.69)	2.50 (1.50 - 3.75)	2.50 (1.25 - 3.58)									
Range	1.00 - 8.00	1.00 - 8.00	1.00 - 5.00									
Categorization of epinephrine interval dosing	14 (38.89 %)	9 (36.00 %)	5 (45.45 %)	0.806	36							
3–5min	21 (58.33 %)	15 (60.00 %)	6 (54.55 %)			—	—			—	—	
< 3min	1 (2.78 %)	1 (4.00 %)	0 (0.00 %)			0.72	0.167 - 3.149	0.656		0.831	0.079, 8.815	0.874
> 5min						NA				NA		

PCPC, Pediatric Cerebral Performance Category.

1 n, population number ( %).

2 Pearson’s Chi-squared test; Fisher’s exact test; Wilcoxon rank sum test.

3 OR, Odds Ratio, CI, confidence interval; min, minutes; OR, odds ratio; CPR, cardiopulmonar resuscitation; SD, Standart deviation; IQR, interquartile range.

## Discussion

This study evaluated the association between epinephrine dosing intervals during pediatric in-hospital cardiopulmonary resuscitation and one-year survival, survival to hospital discharge, and one-year neurological outcomes. Current resuscitation guidelines recommend epinephrine administration every 3–5 min during CPR11. In the studied cohort, although shorter dosing intervals (< 3 min) were associated with higher one-year survival in univariate analyses, this association was not sustained after adjustment for relevant confounders. No association was identified between epinephrine dosing intervals and neurological outcomes.

The association between epinephrine dosing interval and outcomes must be interpreted in the context of cardiopulmonary resuscitation duration. As resuscitation efforts become prolonged, the spacing between epinephrine doses naturally increases, making the dosing interval a partial surrogate for CPR length. This phenomenon has been described in pediatric resuscitation studies, including those by Hoyme et al. (2017)[1] and Ortmann et al. (2023) [19], both of which demonstrated a strong correlation between longer dosing intervals and longer CPR duration.

This study population has a profile similar to other in-hospital pediatric CPR studies conducted in major centers[20] allowing for comparison of the results. The population came from a single quaternary care center in a middle-income country, specialized in treating critically ill pediatric patients, predominantly with complex chronic conditions (95.6 %), who were under CPR and had survived. Survival rates to hospital discharge in children with IHCA have improved over the last decade[2], and higher rates of favorable neurological prognosis among survivors have also been reported [21], as shown in a study by Shimoda-Sakano et al. (2020) [22].

Kienzly et al. (2021) [13], in a retrospective study at a single pediatric center, reported that administering epinephrine at intervals of 2 min or less was associated with increased diastolic pressure during CPR, shorter CPR duration, increased ROSC, and better discharge outcomes with good neurological prognosis. In contrast, in this study, no association was observed with hospital discharge, nor with a favorable neurological outcome at discharge.

Recently, in a multicenter observational study, Kienzly et al. (2024)[12] reported that patients who received at least two doses of epinephrine with intervals of <3 min were not associated with better neurological outcomes; however, they were associated with sustained ROSC and a shorter CPR duration, as shown in the present study, which also did not find an association with neurological outcomes, but with one year survival.

In contrast, Hoyme et al. (2017) [1], in a multicenter retrospective study, demonstrated that administering epinephrine during pediatric IHCA at intervals greater than five minutes was associated with higher in-hospital survival when adjusted for CPR duration [OR = 1.81 (95 % CI 1.26–2.59) and 2.64 (95 % CI 1.53–4.55) for intervals of > 5–8 min and 8–10 min, respectively]. Unlike the unadjusted findings, in which shorter epinephrine intervals were initially associated with improved one-year survival, this association was not sustained after multivariable adjustment. This attenuation likely reflects the strong influence of CPR duration on outcomes, reinforcing the concept that epinephrine dosing interval and resuscitation length are intrinsically interrelated variables. The discrepancy can be attributed to the differences in the study's design, such as the number of cases included, single vs multicenter study, and the different intervals of epinephrine doses. Epinephrine interval doses on pediatric CPR outcomes is complex and challenging to study, which is why this topic remains controversial.

There are also studies in the adult population that have attempted to better clarify the association between epinephrine dosing intervals and outcomes during CPR. For example, a recent meta-analysis[23] on adult CPR did not demonstrate a significant association between epinephrine dose intervals and survival with better hospital outcomes and favorable neurological outcomes. In contrast, the present study found that shorter epinephrine administration intervals were associated with increased one-year survival rates.

Epinephrine is a potent inotropic, chronotropic, and vasopressor agent widely used in CPR [24]. However, the deleterious and cumulative effects of epinephrine, such as arrhythmias and tissue hypoperfusion culminating in neurological damage, seem to be related to the greater number of epinephrine doses, as demonstrated in previous studies [8,10]. In a situation of circulatory collapse, such as CA, distinguishing between beneficial and harmful effects becomes challenging. The development of continuous monitoring methods to guide interventions during CA is crucial. For example, invasive blood pressure measurement is already used in many centers for this purpose. In the present study, however, this parameter could not be analyzed as it is not yet included in the institutional CPR registry.

Although in the present study the CPR outcomes were not adjusted with CA rhythms, this topic remains of interest in resuscitation studies and is related to patient prognosis and outcomes [25]. There is a trend in the literature to analyze symptomatic bradycardia apart from asystole and pulseless electrical activity, as the physiological response of patients with bradycardia and poor perfusion[3] receiving epinephrine can be different. In bradycardia, the precise mechanisms driving ROSC outcomes are not fully understood, but the hemodynamic response during CPR has been speculated, perhaps because CPR is initiated at an earlier stage of the cardiorespiratory collapse process. It is worth noting that bradycardia was the initial rhythm observed in 47.89 % of the cases in this study; therefore, the interpretation of the results should take this aspect into account.

The present study also evaluated other parameters known to interfere with in-hospital CPR prognosis, such as prearrest continuous vasoactive drugs infusion, the number of epinephrine doses, the initial rhythm, and the duration of CPR. Importantly, although these variables were included in the adjusted analyses, their role in the results obtained when studying epinephrine cannot be completely ruled out. Administration of prearrest continuous vasoactive drugs infusion was associated with a lower chance of discharge (OR: 0.17; 95 % CI 0.06–0.40; *p* < 0.001) and lower one-year survival (OR: 0.18; 95 % CI 0.06–0.43; *p* < 0.001), corroborating with other studies that also reported lower survival to hospital discharge [22,26,27,28].

Another relevant topic in CPR is the number of epinephrine doses. A narrative review addressing the use of epinephrine in pediatric CPR[29] revealed an inverse relationship between the number of epinephrine doses and survival, as did other studies [30]. This study revealed the same associations for both survival to discharge and one-year survival (OR: 0.63; 95 % CI 0.46–0.82; *p* = 0.002 and OR: 0.67; 95 % CI 0.49–0.86; *p* = 0.006). Regarding CPR duration, an inverse association was also found, with shorter CPR times associated with a greater chance of survival to discharge (OR 0.87; 95 % CI 0.77–0.96; *p* = 0.01) and one-year survival (OR 0.88; 95 % CI 0.77–0.97; *p* = 0.03). Another point attempted to elucidate the factors associated with longer CPR duration in children with IHCA without success. Understanding the factors that influence the duration of CPR and its relationship with pediatric survival, taking into account the patient’s profile and the hospital environment, can be essential to improve outcomes.

Neurological outcome has been a current topic of investigation [2,4,6,20], and an association with factors such as CPR duration, baseline PCPC, number of epinephrine doses, and prearrest continuous vasoactive drugs infusion has been demonstrated. In the present study, neurological status worsened at one-year survival in 16.6 % of the patients (*p* = 0.01), and no significant association was found with the epinephrine dose interval. Kienzle et al. (2024)[13] obtained similar results related to survival to discharge. In another study, Kienzle et al. (2021)[12] reported favorable discharge outcomes with good neurological prognoses associated with epinephrine intervals. This inconsistency among studies demonstrates the difficulty and complexity in analyzing neurological prognosis after CPR in relation to epinephrine.

It is important to point out that there is currently no adequate tool for assessing neurological prognosis after CPR. Although most studies use the PCPC, there is a movement within the scientific community to find better parameters for evaluating neurological status [18]. This is also a concern of ILCOR in its latest update in 2023 [11].

It is crucial to highlight some limitations of this study. Most limitations are inherent to the observational nature of the study, where causation cannot be established. On the other hand, observational studies are feasible and closely approximate reality, whereas randomized clinical studies in pediatric CPR are challenging, lengthy, expensive, and have limited clinical applicability owing to the rigid exclusion criteria. Another possible limitation is the lack of invasive blood pressure data and the absence of quality CPR parameters. An important aspect to consider is that the study was conducted in a single center, making it difficult to generalize the results, but on the other hand, it has high internal validity and contributes to improvements in CPR within the institution. The population consists almost entirely of patients with preexisting serious conditions, which could be considered a limitation, but, from another point of view, this is precisely the growing profile of hospitalized children. Overall, the present findings should be interpreted as hypothesis-generating rather than definitive, reinforcing the need for cautious interpretation of univariate associations in resuscitation research.

In conclusion, in this cohort, epinephrine dosing interval was not independently associated with one-year survival after adjustment for relevant confounders. The findings underscore the complexity of resuscitation processes and support further investigation in larger, multicenter studies. No relationship was observed between the epinephrine dose interval and one-year neurological prognosis.

## Grant/Funding source

Without institutional involvement, all costs were borne by the authors.

## Data availability

The data that support the findings of this study are available from the corresponding author.

## Conflicts of interest

The authors declare no conflicts of interest.

## References

[1] Hoyme D.B., Patel S.S., Samson R.A., Raymond T.T., Nadkarni V.M., Gaies M.G. (2017). Epinephrine dosing interval and survival outcomes during pediatric in-hospital cardiac arrest. Resuscitation.

[2] Girotra S., Spertus J.A., Li Y., Berg R.A., Nadkarni V.M., Chan P.S. (2013). Survival trends in pediatric in-hospital cardiac arrests: an analysis from get with the Guidelines-Resuscitation. Circ Cardiovasc Qual Outcomes.

[3] Martinez P.A., Totapally B.R. (2016). The epidemiology and outcomes of pediatric in-hospital cardiopulmonary arrest in the United States during 1997 to 2012. Resuscitation.

[4] Reis A.G., Nadkarni V., Perondi M.B., Grisi S., Berg R.A. (2002). A prospective investigation into the epidemiology of in-hospital pediatric cardiopulmonary resuscitation using the international Utstein reporting style. Pediatrics.

[5] Al-Eyadhy A., Almazyad M., Hasan G., AlKhudhayri N., AlSaeed A.F., Habib M. (2023). Outcomes of cardiopulmonary resuscitation in the pediatric intensive care of a tertiary center. J Pediatr Intensive Care.

[6] Shimoda-Sakano T.M., Paiva E.F., Schvartsman C., Reis A.G. (2023). Factors associated with survival and neurologic outcome after in-hospital cardiac arrest in children: a cohort study. Resusc Plus.

[7] Meert K., Telford R., Holubkov R., Slomine B.S., Christensen J.R., Berger J. (2018). Paediatric in-hospital cardiac arrest: factors associated with survival and neurobehavioural outcome one year later. Resuscitation.

[8] López-Herce J., Del Castillo J., Matamoros M., Cañadas S., Rodriguez-Calvo A., Cecchetti C. (2013). Factors associated with mortality in pediatric in-hospital cardiac arrest: a prospective multicenter multinational observational study. Intensive Care Med.

[9] Donnino M.W., Salciccioli J.D., Howell M.D., Cocchi M.N., Giberson B., Berg K. (2014). Time to administration of epinephrine and outcome after in-hospital cardiac arrest with non-shockable rhythms. BMJ.

[10] Finn J., Jacobs I., Williams T.A., Gates S., Perkins G.D (2019). Adrenaline and vasopressin for cardiac arrest. Cochrane Database Syst Rev.

[11] Berg K.M., Bray J.E., Ng K.C., Liley H.G., Greif R., Carlson J.N. (2023). 2023 International consensus on cardiopulmonary resuscitation and emergency cardiovascular care science with treatment recommendations. Circulation.

[12] Kienzle M.F., Morgan R.W., Reeder R.W., Ahmed T., Berg R.A., Bishop R. (2024). Epinephrine dosing intervals are associated with pediatric in-hospital cardiac arrest outcomes: a multicenter study. Crit Care Med.

[13] Kienzle M.F., Morgan R.W., Faerber J.A., Graham K., Katcoff H., Landis W.P. (2021). The effect of epinephrine dosing intervals on outcomes from pediatric in-hospital cardiac arrest. Am J Respir Crit Care Med.

[14] Warren S.A., Huszti E., Bradley S.M., Chan P.S., Bryson C.L., Fitzpatrick A.L. (2014). Adrenaline (epinephrine) dosing period and survival after in-hospital cardiac arrest. Resuscitation.

[15] Grunau B., Kawano T., Scheuermeyer F.X., Drennan I., Fordyce C.B., van Diepen S. (2019). The association of the average epinephrine dosing interval and survival with favorable neurologic status at hospital discharge in out-of-hospital cardiac arrest. Ann Emerg Med.

[16] Nolan J.P., Berg R.A., Andersen L.W., Bhanji F., Chan P.S., Donnino M.W. (2019). Cardiac arrest and cardiopulmonary resuscitation outcome reports: update of the Utstein resuscitation registry template for in-hospital cardiac arrest. Circulation.

[17] Fiser D.H. (1992). Assessing the outcome of pediatric intensive care. J Pediatr.

[18] Topjian A.A., Scholefield B.R., Pinto N.P., Fink E.L., Buysse C.M.P., Haywood K. (2020). P-COSCA (Pediatric Core Outcome Set for Cardiac Arrest) in children. Circulation.

[19] Ortmann L.A., Reeder R.W., Raymond T.T., Brunetti M.A., Himebauch A., Bhakta R. (2023). Epinephrine dosing strategies during pediatric extracorporeal cardiopulmonary resuscitation. Resuscitation.

[20] Sutton R.M., Reeder R.W., Landis W., Meert K.L., Yates A.R., Berger J.T. (2018). Chest compression rates and pediatric in-hospital cardiac arrest survival outcomes. Resuscitation.

[21] Rodríguez-Núñez A., López-Herce J., del Castillo J., Bellón J.M. (2014). RIBEPCI. Shockable rhythms and defibrillation during in-hospital pediatric cardiac arrest. Resuscitation.

[22] Shimoda-Sakano T.M., Schvartsman C., Reis A.G. (2020). Epidemiology of pediatric cardiopulmonary resuscitation. J Pediatr (Rio J).

[23] Wongtanasarasin W., Srisurapanont K., Nishijima D.K. (2023). How epinephrine administration interval impacts the outcomes of resuscitation during adult cardiac arrest. J Clin Med.

[24] Perkins G.D., Ji C., Deakin C.D., Quinn T., Nolan J.P., Scomparin C. (2018). A randomized trial of epinephrine in out-of-hospital cardiac arrest. N Engl J Med.

[25] Morgan R.W., Reeder R.W., Meert K.L., Telford R., Yates A.R., Berger J.T. (2020). Survival and hemodynamics during pediatric cardiopulmonary resuscitation. Crit Care Med.

[26] Rathore V., Bansal A., Singhi S.C., Singhi P., Muralidharan J. (2016). Survival and neurological outcome following in-hospital paediatric cardiopulmonary resuscitation in North India. Paediatr Int Child Health.

[27] Alten J.A., Klugman D., Raymond T.T., Cooper D.S., Donohue J.E., Zhang W. (2017). Epidemiology and outcomes of cardiac arrest in pediatric cardiac ICUs. Pediatr Crit Care Med.

[28] Lee E.P., Chan O.W., Lin J.J., Hsia S.H., Wu H.P. (2022). Risk factors and neurologic outcomes associated with resuscitation in the pediatric intensive care unit. Front Pediatr.

[29] Faria J.C.P., Victorino C.A., Sato M.A. (2020). Epinephrine in pediatric cardiorespiratory arrest: when and how much?. Einstein.

[30] Donoghue A.J., Abella B.S., Merchant R., Praestgaard A., Topjian A., Berg R. (2015). Cardiopulmonary resuscitation for in-hospital events in the emergency department. Resuscitation.

